# Controversies in Parenteral Protein Intake in Preterm Infants

**DOI:** 10.3390/children12060759

**Published:** 2025-06-12

**Authors:** Ira Holla, Pradeep Alur

**Affiliations:** 1 Department of Pediatrics, University of Mississippi Medical Center, Jackson, MS 39216, USA; 2 Hampden Medical Center, Penn State College of Medicine, Hershey, PA 17033, USA; palur@pennstatehealth.psu.edu

**Keywords:** protein, preterm, nutrition

## Abstract

As the limit of viability is extended to lower gestational ages, neonatologists caring for preterm infants must discover the optimal nutritional combination to support postnatal growth. It has been well established that introducing protein soon after birth is associated with improved short-term growth at 36 weeks postmenstrual age and neurodevelopment. However, it remains unclear what the optimal level of protein is for parenteral nutrition at various gestational ages. Several studies have shown possible adverse effects of high-protein delivery in very low birth weight infants. Inborn errors in amino acid metabolism also caution us that higher levels of specific amino acids can harm the growing brain.

## 1. Introduction

Protein, a fundamental macronutrient, is a cornerstone in the nutritional management of preterm infants. As a critical substrate for cellular growth, organ development, and synthesizing essential enzymes and hormones, protein is crucial in supporting the accelerated growth required for preterm infants to achieve catch-up growth comparable to their full-term peers. Moreover, optimal protein intake shortly after birth is closely associated with improved clinical outcomes, including enhanced short-term growth at 36 weeks postmenstrual age, neurodevelopment, and reduced morbidity by increasing protein accrual to support tissue growth, including the preterm brain [1,2,3,4,5].

However, high protein intake may lead to biochemical derangements, such as metabolic acidosis, hyperammonaemia, and refeeding syndrome [6]. Refeeding syndrome in very low birth weight infants is generally seen in the first few days of life after a period of intrauterine malnutrition, such as placental insufficiency. This is characterized by hypophosphatemia, hypercalcemia, hypokalemia, hypomagnesemia, hyperglycemia, and thiamin deficiency. The ProVIDe trial showed that for each 1 g/kg/d increase in IV protein intake, the risk of refeeding syndrome increases by 80% [6]. The ideal amount of protein for infants born prematurely is still unclear [3,6,7].

In utero, the transplacental amino acid passage rate is 3.5–4 g/kg/day, of which a certain fraction is oxidized for energy production [8]. A recent systematic review of high versus low dietary protein intake in neonates born preterm considered protein provided parenterally and enterally together and concluded that high protein may be associated with no longer-term growth benefits and that higher intakes may represent a risk for neonatal metabolism and later neuro-disability [9].

Amino acids provided through different routes, such as parenteral and enteral, can have different biochemical effects. When the amino acids are enterally provided, the splanchnic metabolism increases, leading to higher energy and AA requirements [10]. Moreover, splanchnic tissue may correct any deficient AA absorbed enterally, thus improving the protein quality. This advantage is lost when amino acids are given parenterally, as this route bypasses splanchnic tissue and also leads to lower amino acid requirements. Therefore, our review will focus specifically on parenterally administered amino acids.

## 2. Biochemical Basis of Adverse Effects from Excessive Protein Intake

Branched-chain amino acids (BCAAs), particularly leucine, activate the mechanistic Target Of Rapamycin (mTOR) signaling pathway, enhancing muscle protein synthesis and preventing its breakdown. mTOR is a protein kinase that regulates essential cellular processes, including protein synthesis, autophagy, and glucose homeostasis. Disruptions in mTOR signaling have been linked to the progression of several health issues, such as cancer and diabetes, and may also contribute to the aging process. Conversely, excess BCAAs and their metabolites are associated with neurological dysfunction and acidemias. Excess branched-chain AA (BCAA) concentrations have been linked [11] to impaired growth and development in human and animal studies. The possible mechanisms are related to insulin release, tissue protein synthesis/degradation, catabolism of other branched-chain AAs, and transport of large neutral AAs into tissues, particularly the brain. It is suggested that chronic accumulation of plasma BCAA levels could interfere with insulin signaling via activation of the mTOR/p70S6K pathway [12]. Increased protein intake enhances amino acid oxidation, which helps maintain amino acid homeostasis in the organism. However, as AA cannot be stored, the risk of free radical generation increases [13]. Leucine may compete with phenylalanine and tyrosine, which are important neurotransmitter precursors for transport across the blood–brain barrier, disrupting neurotransmission. Excess of branched-chain amino acids may lead to the accumulation of branched-chain ketoacids. Excess BCAAs also induce lipid peroxidation in the brain and thus induce neurotoxicity. Excess Leucine and branched ketoacids interfere with the Pyruvate Dehydrogenase enzyme and mitochondrial respiratory chain, resulting in energy failure in the brain [14]. Thus, moderation is the key, and finding the optimal requirement of AA is vital.

### 2.1. High Protein and Later Obesity

The first 1000 days of life are vital for body and brain development from conception to age two [15]. This period also represents the best opportunity for preventing obesity and its negative consequences [16,17,18]. Protein plays a fundamental role in supporting healthy growth and development during these early years. The nutrition a child receives in infancy lays the groundwork for their risk of obesity and various health outcomes later in life.

Recent research suggests that a high protein intake during infancy may be linked to an increased body mass index (BMI) and a higher risk of obesity as a child enters adolescence [19,20]. One key mechanism behind this relationship is that excessive protein consumption in infancy can lead to increased insulin secretion and elevated insulin-like growth factor I (IGF-I) levels [21]. This adipogenic hormone surge may contribute to rapid weight gain during this crucial period, setting the stage for obesity later in life. Notably, studies have shown that experiencing swift weight gain in the first two years of life is associated with a threefold increase in the likelihood of becoming obese in later childhood [22].

A meta-analysis of 10 cohort studies conducted in 2012 concluded that each increase in weight gain z-score from birth to one year raises the risk of being overweight or obese in adulthood [23]. To put it differently, keeping an eye on weight gain during a baby’s first year can play a crucial role in preventing obesity later. Managing weight gain during the first year of life could effectively prevent obesity. Melnik proposed that Leucine, being a precursor of fatty acid and cholesterol synthesis, activates mTORC1 besides adipogenesis. It is also suggested that mTORC regulates the hypothalamus by reducing the expression of orexigenic peptides [24]. A higher leucine content in cow’s milk formula than breast milk has been associated with childhood obesity [25]. Thus, early programming by protein intake can affect the later development of obesity [26]. This strongly suggests that we should also pay attention to the amino acid levels we achieve with parenteral nutrition and whether they are comparable to fetal levels.

Therefore, long-term follow-ups of those who received high protein during the neonatal hospital course can clarify whether high-protein recipients are at high risk for obesity. The challenge in feeding preterm infants is that enhancing nutritional delivery to promote weight gain, optimize linear growth, and maximize neurodevelopmental outcomes may increase the risk of obesity [27]. However, avoiding very high protein administration is essential until we have better evidence of its safety.

### 2.2. High Protein and Metabolic Acidosis

Amino acid solutions for preterm infants are usually acidic with an average pH of 5.5 [28]. Acidity helps to administer higher calcium doses to meet the growing needs of preterm infants. Amino acid oxidation following high doses of amino acids may cause metabolic acidosis. A recent French study compared mean protein administration of 2.1, 3.1, and 3.3 g/kg/d in the first week of life in preterm infants with a mean gestational age of 27.5 weeks [29]. The group that received the 3.3 g/kg/d AA dose had significant metabolic acidosis with a mean pH of 7.24 compared to 7.30 in those that received 3.1 g/kg/d. The base deficit was also similarly high in the 3.3 g/kg/d AA group. Several reasons have been suggested for metabolic acidosis in this group: lower rate of urinary ammonia excretion and renal loss of bicarbonate secondary to immature renal function.

Another study reported that metabolic acidosis within the first week of life was associated with a higher likelihood of bronchopulmonary dysplasia (BPD) or death in very low birth weight infants [30]. The adjusted odds ratio for BPD was 3.461, with a 95% CI of 1.325–9.042 in the first week of life with metabolic acidosis. Furthermore, metabolic acidosis in the first week after birth was associated with a higher likelihood of developing bronchopulmonary dysplasia (BPD), with an adjusted odds ratio (aOR) of 3.461 (95% CI 1.325–9.042). This initial period coincides with administering parenteral amino acids (AA). Another study comparing 1.5 g/kg/d of AA to 3 g/kg/d found no metabolic acidosis, and the base deficit was also similar between the groups. The bicarbonate level was 22.8 ± 2.3 mEq/dL for the 1.5 g/kg/d group compared to 22.4 ± 2.4 mEq/dL (*p* = 0.349) [31]. A meta-analysis of high versus standard AA groups described a higher need for acetate administration in the high AA groups than in the standard AA groups [32]. Therefore, carefully monitoring the amount of AA given to infants with very low birth weight may help decrease the risk of metabolic acidosis.

A retrospective cohort study reported that metabolic acidosis within the first few days of life in preterm infants with a mean gestational age of 25 weeks is associated with intraventricular hemorrhage after adjusting for other confounding variables, such as arterial carbon dioxide levels [33].

### 2.3. High Protein and Refeeding Syndrome

American Society for Parenteral and Enteral Nutrition (ASPEN) in 2020 published [34] a definition of refeeding syndrome for adults and pediatric populations as follows ”a measurable reduction in concentrations of one or any combination of phosphorus, potassium, and/or magnesium, or the manifestation of thiamine deficiency, developing shortly (hours to days) after initiation of caloric provision to an individual who has been exposed to a substantial period of undernourishment”. However, a few studies on refeeding syndrome in the neonatal population have used varying definitions but have included one or more of the electrolytes mentioned in the ASPEN definition [6,35,36,37,38]. In this article, we will only consider RS with higher protein administration.

**Figure 1:**
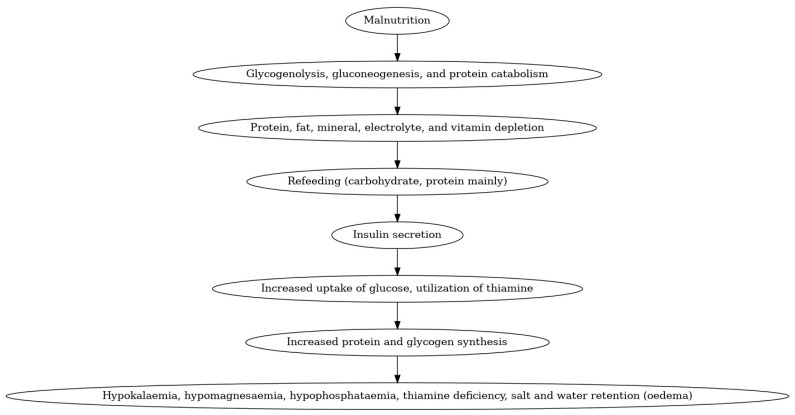
Refeeding syndrome in neonates.

During starvation, the body depletes critical nutrients, particularly potassium and phosphorus. Upon the initiation of refeeding, elevated insulin levels facilitate the transfer of these electrolytes into the cells, resulting in decreased blood concentrations of phosphorus and potassium (Figure 1). This condition may lead to significant complications, including cardiac arrhythmias, muscle weakness, and respiratory failure. Specifically, phosphorus depletion hinders energy production and impairs oxygen transport to tissues, which can pose a life-threatening risk [39]. Typically, placental insufficiency of varied etiology can cause intrauterine growth restriction due to inadequate metabolite transfer. When these small for gestational age infants are initiated on parenteral nutrition with insufficient electrolytes, they may experience RS. The incidence of RS may vary based on the study group’s chosen definition of RS [38]. The reported incidence is between 20% and 90%.

One study noted that the risk of hypophosphatemia (<4 mg/dL) had an OR of 3.13 [95% CI, 2.33–4.21] when protein-containing fluids were administered compared to protein-free fluids [40]. A prospective randomized control trial that compared 3.5 g/kg/d of protein and 2 g/kg/d of protein, noted an incidence of hypophosphatemia in 77% in the former compared to 26% in the latter (*p* = 0.001) [35]. In their prospective observational study, Bonsante et al. investigated three groups of preterm neonates based on their initial amino acid intake: less than 1.5 g/kg/day, 1.5 to 2 g/kg/day, and more than 2 g/kg/day [41]. The incidence of severe hypophosphatemia (defined as levels below 3.1 mg/dL) was recorded as 0% in the group receiving less than 1.5 g/kg/day, 4.6% in the 1.5 to 2 g/kg/day group, and 12.5% in the group receiving more than 2 g/kg/day. The differences among these groups were statistically significant (*p* < 0.001). In another study, severe hypophosphatemia was higher in the high amino acid group than in the low amino acid group, with rates of 19.5% versus 7.3% (*p* = 0.04) [37]. The odds were significantly increased (OR, 18 [95% CI, 6–53.8]) for those who were small for gestational age receiving early high amino acid treatment compared to appropriate for gestational age extremely low birth weight infants.

Clinical Outcomes with RS: hypophosphatemia is associated with a decrease in adenosine triphosphate, which may lead to respiratory muscle hypotonia with a possible longer duration of mechanical ventilation. However, a few studies that focused on this aspect could not find a statistical difference in the duration of mechanical ventilation [42,43]. Similarly, the association between hypophosphatemia and sepsis or bronchopulmonary dysplasia is also inconclusive [44,45]. The association between hypophosphatemia and intraventricular hemorrhage is equivocal [45,46]

The evidence thus suggests that clinicians should be mindful of this potential complication with higher amino acid dosage and proceed cautiously.

## 3. Studies Looking at Varying Protein Administration [Table 1]

Thureen et al. [47] studied the efficacy and safety of increased protein intake in 28 infants with birth weight ≤1300 g. They divided them into a High Amino Acid group (HAA) receiving 3 g/kg/day of amino acids and a Low Amino Acid group (LAA) receiving 1 g/kg/d of amino acids. They compared plasma AA levels of essential and non-essential AA in the HAA group to human fetal AA levels obtained through umbilical cord sampling from a study by Cetin et al. [8]. They found that the HAA group had significantly higher Leucine, Isoleucine, Phenylalanine, Methionine, Proline, Arginine, and Ornithine levels. It is well known that high levels of certain AA are associated with microcephaly, such as serine [48], glycine [49], asparagine [50], phenylalanine, and branched-chain AA [51]. At a dosage of 3 g/kg/d, which was deemed the safe limit for amino acid administration, the authors observed elevated concentrations of branched-chain amino acids. Therefore, it is concerning that exceeding the AA threshold to over 4 g/kg/d could push these AA levels to higher levels, which may be harmful. Hence, cautious administration is critical.

**Table 1:** Summary of findings from studies looking at varying protein administration.

Study	Finding
[47]	Higher Amino Acid (HAA, receiving 3 g/kg/day of amino acids as compared to Low Amino Acid group (LAA) receiving 1 g/kg/d of amino acids) group had significantly higher plasma Leucine, Isoleucine, Phenylalanine, Methionine, Proline, Arginine, and Ornithine levels.
[51]	Significantly lower z scores for head circumference in the high AA group (which started with a dose of 2 g/kg/day of parenteral AA on DOL 1 and was increased by 1 g/kg/day to a maximum of 4 g/kg/day, which continued till DOL 7, as compared to the Standard AA group which received 0.5 g/kg/day of parenteral AA starting the day of life (DOL) 1, increased by 0.5 g/kg/day daily to a maximum of 3 g/kg/day, which continued till DOL7) at 6, 12, 18, and 24 months
[52]	High AA group with significantly higher levels of individual amino acids as compared to levels in second-trimester fetuses.
[53]	Propensity score-matched analysis of infants enrolled in the EPIPAGE-2 study-primary outcome of Full Scale IQ greater than −1 SD (i.e., ≥93 points) at age 5 years was more frequent in the exposed (high amino acid intake (3.51–4.50 g/kg/d) 7 days after birth) vs. the unexposed group (243 infants [61.4%] vs. 206 infants [54.4%], respectively; odds ratio [OR], 1.33 [95% CI, 1.00–1.71]).
[54]	Significantly lower head circumference growth velocity on postnatal day 28 in High AA group (3 g/kg/day of parenteral amino acids on day 1, and the dose increased to 4 g/kg/day the next day) as compared to Low Amino Acid group (receiving 1 g/kg/day of parenteral amino acids on day 1 and dose increased by 1 g/kg every day till a maximum of 4 g/kg/day)
[55]	Moderate to severe neuro disability was more common in the intervention group who received more than 1 g per day of parenteral protein over the first four postnatal days and more than a mean of 3.5 g per kilogram per day on postnatal days 3, 4, and 5 as compared to the control group (adjusted relative risk, 1.95; 95% CI, 1.09 to 3.48)

Blanco et al. [51] investigated the effect of early and high amino acid supplementation on growth and neurodevelopmental outcomes in ELBW infants. The study had two groups: the standard AA group and the high AA group. The standard AA group received 0.5 g/kg/day of parenteral AA starting the day of life (DOL) 1. The AA was increased by 0.5 g/kg/day daily to a maximum of 3 g/kg/day, which continued till DOL7, and the high AA group, which started with a dose of 2 g/kg/day of parenteral AA on DOL 1 and was increased by 1 g/kg/day to a maximum of 4 g/kg/day, which continued till DOL 7. After DOL 7, both groups received 3.5 g/kg/day until total parenteral nutrition (TPN) was weaned. These infants were followed, and growth was assessed at 6, 12, 18, and 24 months. They found significantly lower z scores for head circumference in the high AA group at each time point. This difference was more pronounced in males as compared to females. They hypothesized that disproportionate concentrations of branched-chain amino acids might play a role in the causation of microcephaly observed in association with high AA in their study.

Another study by Blanco et al. [52] compared plasma amino acid concentrations between infants given standard intravenous AA supplementation (standard AA) or an early and high supplementation regimen (early and high AA). The standard AA group received 0.5 g/kg/d intravenous AA starting between the first 24 to 36 h of life, with increases of 0.5 g/kg/d every 24 h to a maximum of 3.0 g/kg/d and continued until the seventh day of life (DOL). The early and high AA group received 2.0 g/kg/d of intravenous AA soon after enrollment and within the first 24 h of life, with 1 g/kg/d increases every 24 h up to a maximum of 4 g/kg/d and continued until DOL 7. They found significant differences in concentrations for all AAs except the nonessential AAs Glu, Asn, Gly, Gln, Ala, and Tyr and the conditionally essential AA Cys between the standard and the early and high AA groups. The mean concentration of BCAA-Leucine on day of life 3 was 95.9 µmol/L in the standard versus 239.4 µmol/L in the early high group. Furthermore, compared with levels of individual amino acids in second-trimester fetuses, they found that the early and high AA group had higher levels than the second-trimester fetuses. The fetal leucine levels during the second trimester were 77.4 ± 3.3 µmol/L compared to 239.4 µmol/L in preterm infants with early high AA administration. Similarly, fetal Isoleucine levels were 39.8 ± 2.7 µmol/L compared to 212.8 µmol/L in the preterm with early high AA administration. The levels of isoleucine are almost five times higher with high AA administration.

Morel et al. [53] conducted a propensity score-matched analysis of infants enrolled in the EPIPAGE-2 study [56] comparing infants born at less than 30 weeks gestation with high amino acid intake (3.51–4.50 g/kg/d) 7 days after birth with infants who did not. Full-scale IQ (FSIQ) was assessed at age 5 years, with the primary outcome being an FSIQ score greater than −1 SD (i.e., ≥93 points) at age 5 years. Their analysis found that the primary outcome was more frequent in the exposed vs. the unexposed group (243 infants [61.4%] vs. 206 infants [54.4%], respectively; odds ratio [OR], 1.33 [95% CI, 1.00–1.71]). The study recommended a cautious approach to administering higher amounts of AA during the initial days of life in preterm infants.

Similarly, Balasubramanian et al. [54] studied the impact of higher protein delivery in VLBW infants. Protein delivery was classified into two groups—(1) the Low Amino Acid (AA) group receiving 1 g/kg/day of parenteral amino acids on day 1 and dose increased by 1 g/kg every day till a maximum of 4 g/kg/day. (2) The High AA group received 3 g/kg/day of parenteral amino acids on day 1, and the dose increased to 4 g/kg/day the next day. They measured postnatal growth on the day of life 28. The head circumference gain (in cm/week) (HGV) was significantly less in the high AA group. In the low AA group, the mean HGV was 0.625 cm/week versus 0.25 cm/week in the high AA group. They hypothesized this was due to a low ratio of calories from non-protein sources to calories from protein (CNR) in the higher amino acid group. It is important to be aware that sufficient calories from non-protein sources are necessary; otherwise, amino acids will be metabolized to supply energy.

For optimal amino acid utilization, at least 30–40 kcal should be provided per 1 g of protein. ESPGHAN recommended providing more than 65 kcal/kg/d of non-protein energy when administering 2.5 to 3.5 g/kg/d of amino acids in preterm infants [57]. When non-protein calorie intake is unduly low, amino acid blood levels may become elevated as they may not be oxidized and metabolized effectively through the urea cycle.

The other studies that attempted to look at the effect of high vs. low protein intake were unable to achieve higher protein intake for their intervention groups. For example, the study by Burattini et al. [58] investigated the effect of increased protein intake from a standard of 2.5 g/kg/day (Standard Amino Acid group, SAA) to 4 g/kg/day (High Amino Acid group, HAA) in 131 infants with a birth weight between 500 g and 1249 g and found no significant difference in head circumference between the two groups at 36 weeks corrected gestational age (CGA) and at discharge. However, after reviewing their data, it is apparent that the HAA group did not receive the intended amount of protein; instead, it received less than 3.5 g/kg/day of protein, which was within the acceptable limit. Similarly, Morgan et al. [59] aimed to study the effect of increased protein intake in infants <29 weeks GA and <1200 g. They divided the infants into the Control group (receiving 2.8 g/kg/day of protein) and the SCAMP (Standardized, Concentrated with Added Macronutrients Parenteral) group (receiving 3.8 g/kg/day of protein). They found a significant increase in head circumference in the SCAMP group at 28 days of life, an effect that persisted at 36 weeks of CGA. However, a review of their data indicated that the SCAMP group did not receive the intended 3.8 g/kg/day of protein at any point, receiving a maximum of 3.2 g/kg/day, which is still within the acceptable range for protein provision. Therefore, it is essential to ascertain whether the intended and actual provision of protein was consistent across the studies to understand the clinical implications.

Bloomfield and colleagues encountered interesting observations [55] in their multicenter, double-blind, parallel-group, randomized, placebo-controlled trial investigating the effect of an additional 1 g per day of protein administration for the first 5 days of life in infants with birth weight less than 1000 g on neurodevelopmental outcomes at 2 years of age. Compared with the placebo group, the intervention group received more than 1 g per day of parenteral protein over the first four postnatal days and more than a mean of 3.5 g per kilogram per day on postnatal days 3, 4, and 5. While they found no significant difference between the control group and the intervention group in terms of their primary outcome, which was survival without neuro disability at 2 years of age; however, they did find that moderate to severe neuro disability was more common in the intervention group (adjusted relative risk, 1.95; 95% CI, 1.09 to 3.48). However, the lower protein-to-energy ratio of 15 may be responsible for the poor outcomes. Another recent study that compared low non-protein energy to a gram of protein (NPKcal:g) administration through parenteral nutrition noted that a ratio of >21:1 of NPKcal:g may be necessary to prevent oxidation of amino acids administered [60].

The differences between enteral and parenteral protein intake must be considered, which may determine the optimal intake for each route. The enteral nutrition undergoes first-pass metabolism in the liver, which is bypassed in parenteral nutrition [10]. Furthermore, most studies looking at the composition of parenteral protein intake are performed in the first few weeks of life. In contrast, parenteral nutrition is a bridge to enteral nutrition, whereas studies looking at enteral protein intake typically enroll older infants when enteral feeds are fully established. Therefore, enteral and parenteral nutrition studies examine different hepatic and renal developmental stages. This may also explain higher tolerance for enteral proteins than parenteral protein intake. In addition, splanchnic metabolism and amino acid turnover are much greater during enteral nutrition than during parenteral. Therefore, due to the above reasons, protein requirements and tolerance may be greater during enteral nutrition than parenteral nutrition; thus, the two cannot be compared.

## 4. Current Recommendation [Table 2]

The American Society for Parenteral and Enteral Nutrition published guidelines [61] for parenteral nutrition in preterm infants. The recommendation is to administer parenteral amino acids at 3–3.5 g/kg/day. It also emphasizes that evidence does not show clear neurodevelopmental benefits for doses above 3.5 g/kg/day, and higher doses may pose risks.

**Table 2:** Recommendations regarding parenteral protein intake in preterm infants.

Institution	Recommendation	
American Society for Parenteral and Enteral Nutrition [61]	Parenteral AA doses at a minimum of 3 g/kg/day without increasing beyond 3.5 g/kg/day.	
European Society for Pediatric Gastroenterology, Hepatology and Nutrition (ESPGHAN) [57]	Parenteral AA intake ranging between 2.5 and 3.5 g/kg/day, accompanied by non-protein calorie intake of 65 kcal/kg/day.	
American Academy of Pediatrics Committee on Nutrition [62]	<1000 g birth weight/kg/day	1000–1500 g birth weight/kg/day
Starting Phase	1.5–2.5 g	3.0–3.5 g
Stable Phase	3.0–3.5 g	3.0–3.5 g

The European Society for Pediatric Gastroenterology, Hepatology and Nutrition (ESPGHAN) published guidelines in 2018 on parenteral amino acids [57], which recommended parenteral amino acid intake in preterm infants ranging between 2.5 and 3.5 g/kg/day accompanied by non-protein calorie intake of 65 kcal/kg/day. These guidelines contrast with their previous recommendations published in 2010 [63] to provide 3.5–4.5 g/kg/day of protein from postnatal day 2 onwards. This change in recommendations stemmed from mounting evidence that excessive protein intake may harm the developing preterm infant.

Conclusion: Therefore, limiting the AA provision through parenteral nutrition in extremely low birth weight infants is prudent until more extensive studies confirm their safety. It will be interesting to investigate whether the AA profile in utero exhibits any sex-specific differences akin to the composition of human breast milk [64]. Human milk studies have shown that preterm human milk may contain higher energy and fat in breast milk for male infants than for female preterm infants. Body composition varies between male and female preterm infants at birth, and it changes when formula is provided instead of the mother’s milk. Consequently, fetal cord sampling studies can shed light on this critical issue.

## Data Availability

No new data were created or analyzed in this study. Data sharing is not applicable to this article.
